# Changes in metabolic syndrome and risk of psoriasis: a nationwide population-based study

**DOI:** 10.1038/s41598-021-03174-2

**Published:** 2021-12-15

**Authors:** Hyun Ji Lee, Kyung Do Han, Hae Eun Park, Ju Hee Han, Chul Hwan Bang, Young Min Park, Ji Hyun Lee

**Affiliations:** 1 Department of Dermatology, Seoul St. Mary’s Hospital, College of Medicine, The Catholic University of Korea, Seoul, Korea; 2 Department of Dermatology, Yeouido St. Mary’s Hospital, College of Medicine, The Catholic University of Korea, Seoul, Korea; 3 Department of Statistics and Actuarial Science, Soongsil University, Seoul, Korea

**Keywords:** Diseases, Risk factors

## Abstract

Metabolic syndrome (MetS) is associated with psoriasis, but it remains unclear whether risk of psoriasis remains in patients whose MetS diagnosis changes. To assess the relationship between risk of psoriasis and changes in MetS components. We obtained data from the National Health Insurance Service of Korea and divided the participants into four groups: individuals without MetS (control); individuals with MetS in 2009, but without MetS in 2012 (pre-MetS); individuals without MetS in 2009, but with newly diagnosed MetS in 2012 (post-MetS); and individuals with MetS during the 2009–2012, period (continuous-MetS). We calculated the risk of psoriasis for each group. Risk of psoriasis was similar in the control and pre-MetS groups but was significantly higher in the post-MetS group (hazard ratio [HR], 1.08; 95% confidence interval [CI], 1.04–1.12) and in the continuous-MetS group (HR, 1.11; 95% CI, 1.07–1.15) than in the control group. Among MetS components, waist circumference showed the strongest association with psoriasis, followed by high-density lipoprotein and triglyceride levels. Risk of psoriasis was higher in patients with continuous- or post-MetS than in those with pre-MetS (regardless of prior MetS status).

## Introduction

Psoriasis is a chronic, immune-mediated skin disease that affects an estimated 125 million people worldwide^1^. The pathophysiology of psoriasis involves a variety of inflammatory cells that secrete cytokines, thereby stimulating the activity of myeloid dendritic cells. The predominant feature of psoriasis involves IL-23-mediated activation of the Th17 pathway^2^. Genetic factors play important roles in the development of psoriasis, and environmental factors can exacerbate the disease. Psoriasis is a disease of the skin and joints, as well as a systemic inflammatory process associated with a range of comorbidities, such as cardiovascular disease, inflammatory bowel disease, metabolic syndrome, and diabetes^3,4^. Metabolic syndrome (MetS) includes hyperglycemia, atherogenic dyslipidemia, elevated blood pressure, and abdominal obesity^5,6^, which are defined as Mets components in this study, and increases the risks of cardiovascular disease and all-cause mortality^7^.

During the past two decades, studies concerning the relationship between MetS and psoriasis have been conducted^8^. In one nationwide population-based study, MetS was positively associated with an increased rate of psoriasis, and this trend became more pronounced in individuals with more MetS components^9^. Furthermore, a prospective study showed that MetS (especially its obesity component) was associated with an increased risk of incident psoriasis^10^. There are many studies concerning the association between the two diseases, but none have investigated whether the risk of psoriasis is affected by changes in MetS diagnosis.

Here, we assessed the associations between risk of psoriasis and changes in MetS components using data from the Korean National Health Insurance Service (KNHIS) database.

## Materials and methods

### Data source

We extracted data from the National Health Claims database of the KNHIS^11^. This database represents the entire Korean population and maintains data comprising inpatient and outpatient medical service records, diagnostic and procedural codes, prescribed medication claims, and patient demographics. All Korean nationals receive a unique identification number at birth; accordingly, their health care records cannot be duplicated or omitted. The KNHIS uses the standard codes of the International Statistical Classification of Diseases and Related Health Conditions, 10th revision (ICD-10). The KNHIS also provides general health examinations and a cancer-screening program. Therefore, all insured Koreans and their dependents enjoy free age-relevant health examinations. We ensured that the data extracted from the KNHIS were complete and anonymized, and the Institutional Review Board of the KNHIS approved our request for these data (NHIS-2019–1-075). In addition, the Ethics Committee of Seoul St. Mary's Hospital, at The Catholic University of Korea, approved our study design and waived the requirement for informed consent from the participants (KC18ZESI0639). All methods were performed in accordance with the relevant guidelines and regulations by including a statement in the methods section.

### Study design and population

We included data from 5,644,324 adults who underwent health examinations including measurement of MetS components in 2009 and in 2012. We used the following definition of MetS in this study (in accordance with the revised criteria of the National Cholesterol Education Program Adult Treatment Panel III)^12^: MetS is present in any individual with three or more of the five MetS components (waist circumference ≥ 90 cm for men and ≥ 85 cm for women, in accordance with the Korean Society for the Study of Obesity cut-off point for abdominal obesity^13^; triglyceride [TG] level ≥ 150 mg/dL or use of medication for elevated TG; high-density lipoprotein [HDL] cholesterol level < 40 mg/dL for men and < 50 mg/dL for women or use of medication to reduce LDL-cholesterol; systolic blood pressure ≥ 130 mmHg or diastolic blood pressure ≥ 85 mmHg or use of antihypertensive medication; fasting glucose level ≥ 100 mg/dL or use of medication for elevated glucose level). In order to accurately evaluate the risk of psoriasis according to changes in MetS, only those newly diagnosed with psoriasis in 2009–2012 were included. For this purpose, among the subjects diagnosed with psoriasis in 2009, subjects who had already been diagnosed with psoriasis in 2008, before 2009, were excluded. We divided the participants into four groups: individuals without MetS in both 2009 and 2012 (control); individuals with MetS in 2009, but not in 2012 (pre-MetS); individuals without MetS in 2009, but with new onset diagnosis in 2012 (post-MetS); and individuals with MetS during the 2009–2012 period (continuous-MetS). We assessed the relationships between MetS components (waist circumference, blood pressure, fasting glucose, and TG and HDL levels) and psoriasis for each group. We tracked the data for all study participants (their health care records) during the 4-year period from 2009 to 2012 to identify patients who developed psoriasis (ICD-10: L40).

### Clinical, laboratory, and anthropometric measurements

The KNHIS regular medical heath examination program includes information concerning variables that can alter the general health status. The following data were obtained in this study: sex, age, smoking and alcohol consumption statuses, and physical activity. Detailed smoking status, alcohol consumption, and physical activity (including the amounts and frequencies) were obtained from health examinations. We classified individuals as non-smokers, ex-smokers, and current smokers based on smoking status. We defined alcohol consumption habits using three categories: abstinence (no alcoholic drinks consumed within the past year), moderate alcohol consumption (< 30 g pure alcohol per day), and heavy alcohol consumption (≥ 30 g pure alcohol per day). We classified individuals’ physical activity as vigorous-moderate or absent. Vigorous-moderate physical activity was defined as ≥ 1 day per week of moderate or vigorous intensity exercise. Venous blood samples for measurement of fasting glucose, lipid profiles, and liver enzyme levels were obtained at each health examination after an overnight fast. Body mass index (BMIs) was calculated based on participant weight (in kilograms) divided by the square of height (in meters); these measurements were performed during the health examinations. The health examinations also included measurements of systolic and diastolic blood pressures and of waist circumference^14^.

### Statistical analysis

We analyzed continuous variables using analysis of variance (ANOVA) and dichotomous variables using the χ^2^ test to characterize study population characteristics in the four groups. We analyzed variables with skewed distributions after logarithmic transformation. The data are shown as means ± standard deviations (continuous variables), numbers and percentages (dichotomous variables), or geometric means and 95% confidence intervals (CIs) (continuous variables with skewed distributions). We performed univariate and multivariate Cox proportional hazard regression analyses to evaluate the associations of MetS component changes with risk of psoriasis. The multivariate model included age, sex, smoking status, alcohol consumption, physical activity, and BMI. The assumptions of the proportional hazard model were confirmed using Schoenfeld residual plot and log–log survival function plot analyses. The overall mean time of follow-up was 1.40 ± 0.4 years, and 80,342 individuals were lost to follow-up. We used log-rank tests to analyze the hazard ratios (HRs) for psoriasis for each group. All statistical analyses were performed using SAS software (version 9.4, SAS Institute, Cary, NC, USA). *P* values < 0.05 were considered statistically significant.

## Results

### Characteristics of the study population

We compared the results of health examinations in 2009 and 2012, among the four groups (control group, n = 3,439,976; pre-MetS group, n = 430,044; post-MetS group, n = 752,360, and continuous-MetS group, n = 1,021,944). The average ages of each group were 46.91 ± 12.28 in the control group, 54.22 ± 12.62 in the pre-MetS group, 53.41 ± 12.63 in the post-MetS group, and 58.86 ± 12.04 in the continuous-MetS group. The proportions of males in each group were 58.0% in the control group, 63.7% in the pre-MetS group, 61.0% in the post-MetS group, and 53.8% in the continuous-MetS group. Table 1 shows total patient demographics by group.

**Table 1:** Characteristics of the study population.

Group	Control	Pre-MetS	Post-MetS	Continuous-MetS	*p*
Number	3,439,976	430,044	752,360	1,021,944	
**Age (year)**	46.91 ± 12.28	54.22 ± 12.62	53.41 ± 12.63	58.86 ± 12.04	< 0.01
20–39 (%)	1,051,997 (30.58)	57,713 (13.42)	117,586 (15.63)	70,411 (6.89)	
40–64 (%)	2,073,796 (60.29)	277,203 (64.46)	484,489 (64.4)	603,283 (59.03)	
≥ 65 (%)	314,183 (9.13)	95,128 (22.12)	150,285 (19.98)	348,250 (34.08)	
**Sex (Masculine)** (%)	1,994,779 (57.99)	273,870 (63.68)	458,596 (60.95)	549,278 (53.75)	< 0.01
**Smoking**					< 0.01
Non-smoker (%)	2,005,130 (58.32)	229,903 (53.48)	410,512 (54.58)	613,413 (60.04)	
Ex-smoker (%)	594,935 (17.3)	89,452 (20.81)	151,765 (20.18)	197,190 (19.3)	
Current smoker (%)	838,113 (24.38)	110,492 (25.7)	189,846 (25.24)	211,010 (20.65)	
**Alcohol consumption**					< 0.01
Abstinence (%)	1,634,380(47.59)	217,310 (50.59)	381,161 (50.73)	601,862 (58.96)	
Moderate (%)	1,610,960 (46.91)	181,200 (42.19)	313,829 (41.77)	350,634 (34.35)	
Heavy (%)	188,808 (5.5)	31,001 (7.22)	56,415 (7.51)	68,277 (6.69)	
**Physical activity (vigorous-moderate)** (%)	2,088,458 (60.75)	250,249 (58.23)	424,278 (56.42)	533,465 (52.23)	< 0.01
**Household income (low)** (%)	666,885 (19.39)	92,797 (21.58)	164,967 (21.93)	236,763 (23.17)	< 0.01
**Place (urban)** (%)	1,531,175 (44.51)	184,593 (42.92)	325,629 (43.28)	442,651 (43.31)	< 0.01
**Height** (cm)	165.08 ± 8.91	164.28 ± 9.62	164.11 ± 9.65	162.21 ± 9.9	< 0.01
**Weight** (kg)	62.69 ± 10.77	66.89 ± 11.8	68.39 ± 12.42	69.12 ± 12.89	< 0.01
**Systolic blood pressure** (mmHg)	118.42 ± 12.99	124.32 ± 13.36	128.68 ± 13.4	130.11 ± 14.14	< 0.01
**Diastolic blood pressure** (mmHg)	74.21 ± 9.07	77.39 ± 9.17	80.03 ± 9.48	79.79 ± 9.77	< 0.01
**Fasting glucose** (mg/dL)	92.83 ± 14.15	99.46 ± 22.99	104.89 ± 22.66	113.95 ± 32.58	< 0.01
**Waist circumference** (cm)	77.94 ± 7.98	82.92 ± 7.63	85.28 ± 7.93	87.51 ± 8.21	< 0.01
**BMI** (kg/m^2^)	22.9 ± 2.75	24.65 ± 2.87	25.24 ± 3.02	26.11 ± 3.18	< 0.01
**Total cholesterol** (mg/dL)	194.29 ± 33.22	202.03 ± 35.71	203.64 ± 40.39	195.83 ± 41.85	< 0.01
ALT* (mg/dL)	20.53 (20.52–20.54)	23.91 (23.88–23.95)	26.92 (26.89–26.95)	27.21 (27.18–27.24)	< 0.01
AST* (mg/dL)	23.54 (23.54–23.55)	25.39 (25.37–25.42)	26.76 (26.74–26.78)	27.18 (27.16–27.2)	< 0.01
GTP* (mg/dL)	24.47 (24.46–24.49)	31.01 (30.94–31.07)	35.17 (35.11–35.22)	35.62 (35.57–35.67)	< 0.01

### Risk of psoriasis according to changes in diagnosis of MetS or its components

Table 2 shows differences in risk of psoriasis based on changes in MetS from 2009 to 2012. Compared with the control group, the risk of psoriasis did not increase significantly in the pre-MetS group (HR, 1.00; 95% CI, 0.96–1.05) but did increase significantly in the post-MetS (HR, 1.08; 95% CI, 1.05–1.12) and continuous (HR, 1.11; 95% CI, 1.07–1.15) groups.

**Table 2:** Multivariable HRs of psoriasis risk according to MetS diagnosis changes (CI, confidence interval; HR, hazard ratio). Values are expressed as HRs (95% CIs) based on Cox proportional hazard regression analysis. Model 1: adjusted for age and sex. Model 2: adjusted for age, sex, smoking, alcohol consumption statuses, physical activity, and BMI. ^a^per 1,000 person-years. BMI, body mass index; CI, confidence interval; HR, hazard ratio; MetS, metabolic syndrome.

	n	Cases	Person-years	Incidence rate^a^	HR (95% CI)	
					Model 1	Model 2
**Metabolic syndrome**						
Control	3,439,976	15,149	4,792,187.9	3.16	1 (ref.)	1 (ref.)
Pre-MetS	430,044	2,187	608,733.5	3.59	1.01 (0.96–1.06)	1.00 (0.96–1.05)
Post-MetS	752,360	4,055	1,057,355.48	3.84	1.10 (1.06–1.14)	1.08 (1.05–1.13)
Continuous-MetS	1,021,944	6,113	1,464,007.41	4.18	1.13 (1.10–1.17)	1.11 (1.07–1.15)

Table 3 shows differences in risk of psoriasis based on changes in MetS components criteria from 2009 to 2012. For the waist circumference criterion, after adjusting for age, sex, smoking, alcohol consumption, exercise, and BMI, we found significant increases in risk of psoriasis in the pre-MetS (HR, 1.05; 95% CI, 1.00–1.11), post-MetS (HR, 1.09; 95% CI, 1.04–1.14), and continuous-MetS (HR, 1.15; 95% CI, 1.10–1.20) groups compared with the control group. For the TG criterion, the pre-MetS (HR, 0.99; 95% CI, 0.95–1.03) group had a risk similar to that of the control group, but both the post-MetS (HR,1.09; 95% CI, 1.05–1.13) and continuous-MetS (HR, 1.09; 95% CI, 1.05–1.12) groups had higher risks than the control group. For the HDL criterion, pre-MetS (HR, 1.06; 95% CI, 1.02–1.11), post-MetS (HR, 1.08; 95% CI, 1.04–1.12), and continuous-MetS (HR, 1.10; 95% CI, 1.06–1.14) groups all had higher risks of psoriasis than the control group. For the blood pressure criterion, we found a decreased risk of psoriasis in both the pre-MetS and continuous-MetS groups, but no significant differences in the post-MetS group compared with the control group. Finally, for the fasting glucose criterion, we found similar risks in all groups.

**Table 3:** Multivariable HRs of psoriasis risk according to MetS component diagnosis changes (CI, confidence interval; HR, hazard ratio). Values are expressed as HRs (95% CIs) based on Cox proportional hazard regression analysis. Model 1: adjusted for age and sex. Model 2: adjusted for age, sex, smoking, alcohol consumption statuses, physical activity, and BMI. ^a^per 1,000 person-years. BMI, body mass index; CI, confidence interval; HR, hazard ratio; MetS, metabolic syndrome.

	n	Cases	Person-years	Incidence rate^a^	HR (95% CI)	
					Model 1	Model 2
**Waist circumference criterion**						
Control	4,088,699	18,851	5,727,299.33	3.29	1 (ref.)	1 (ref.)
Pre-MetS	364,325	1,977	517,279	3.82	1.05 (1.00–1.10)	1.05 (1.00–1.11)
Post-MetS	497,596	2,607	697,897.52	3.74	1.09 (1.046–1.14)	1.09 (1.04–1.14)
Continuous-MetS	693,704	4,069	979,808.44	4.15	1.15 (1.11–1.19)	1.15 (1.10–1.20)
**Blood pressure criterion**						
Control	2,409,678	10,693	3,349,388.08	3.19	1 (ref.)	1 (ref.)
Pre-MetS	626,555	2,976	877,496.73	3.39	0.97 (0.93–1.01)	0.96 (0.92–0.99)
Post-MetS	803,418	3,944	1,126,354.87	3.50	0.99 (0.96–1.03)	0.98 (0.94–1.01)
Continuous-MetS	1,804,673	9,891	2,569,044.62	3.85	0.98 (0.96–1.01)	0.97 (0.94–0.99)
**Fasting glucose criterion**						
Control	3,027,414	13,871	4,242,530.62	3.27	1 (ref.)	1 (ref.)
Pre-MetS	632,259	3,080	884,471.37	3.48	0.99 (0.96–1.03)	0.99 (0.95–1.03)
Post-MetS	895,509	4,498	1,256,739.64	3.58	1.01 (0.98–1.05)	1.00 (0.97–1.04)
Continuous-MetS	1,089,142	6,055	1,538,542.66	3.94	1.03 (0.99–1.06)	1.01 (0.98–1.05)
**Triglyceride criterion**						
Control	2,821,098	12,422	3,941,399.88	3.15	1 (ref.)	1 (ref.)
Pre-MetS	586,456	2,876	830,112.1	3.46	1.01 (0.96–1.05)	0.99 (0.95–1.03)
Post-MetS	842,109	4,427	1,181,705.98	3.74	1.11 (1.07–1.15)	1.09 (1.05–1.13)
Continuous-MetS	1,394,661	7,779	1,969,066.33	3.95	1.12 (1.09–1.15)	1.09 (1.05–1.12)
**HDL cholesterol criterion**						
Control	3,311,289	14,804	4,599,182.21	3.22	1 (ref.)	1 (ref.)
Pre-MetS	549,260	2,800	778,582.81	3.60	1.08 (1.03–1.12)	1.06 (1.02–1.11)
Post-MetS	818,734	4,373	1,154,553.07	3.79	1.10 (1.06–1.14)	1.08 (1.04–1.12)
Continuous-MetS	965,041	5,527	1,389,966.2	3.98	1.13 (1.09–1.16)	1.1 (1.07–1.15)

### Relationship between changes in the number of MetS components at first and second visits and the risk of psoriasis

**Figure 1:**
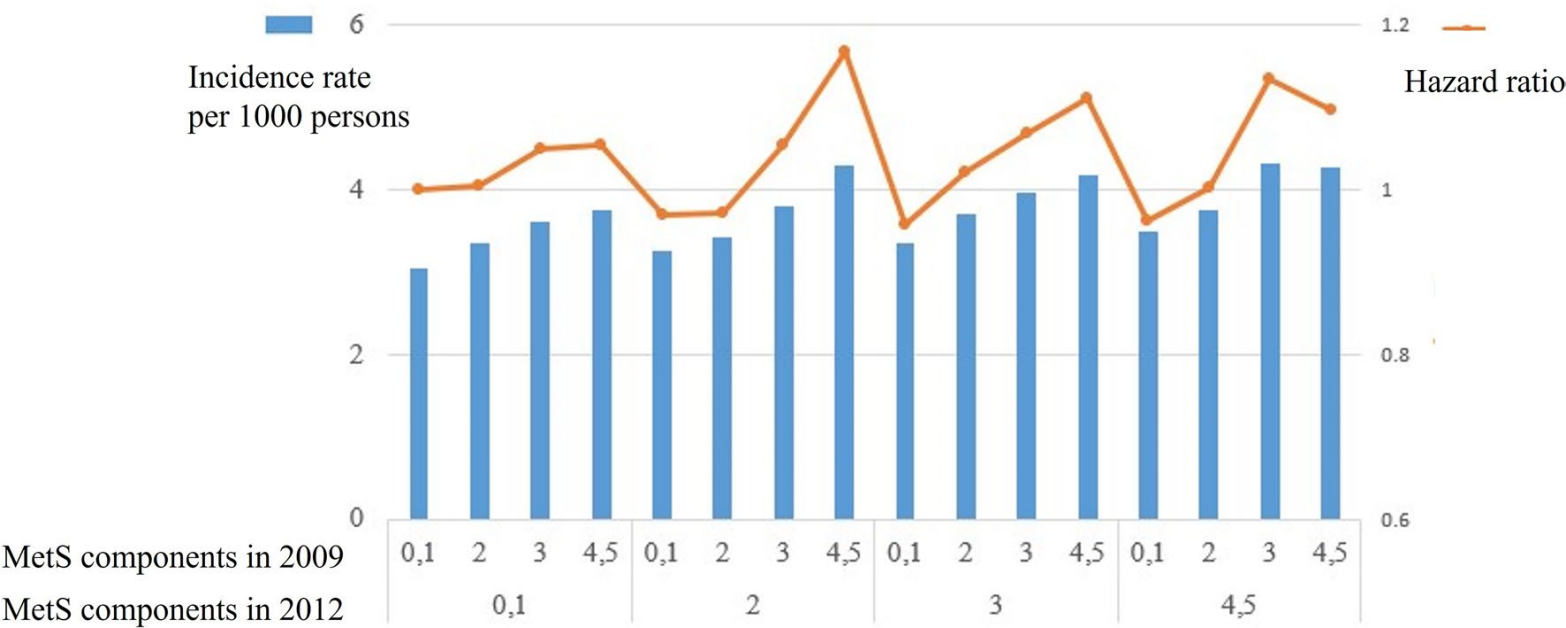
Relationship between change in the number of MetS components from first to second visits and the risk of psoriasis. The lower row of the x-axis represents the number of MetS components satisfied in 2009, and the upper row of the x-axis represents the number of MetS components satisfied in 2012. The blue bar graph with left y-axis represents incidence rate per 1000 persons with psoriasis. The orange line graph and right y-axis represent HR. Regardless of the number of MetS components in 2009, the incidence rate and HR of psoriasis increased according to number of MetS components in 2012.

Next, we plotted our data to visualize the risk of psoriasis according to changes in the numbers of MetS components from 2009 to 2012 (Fig. 1). Regardless of the number of MetS components in 2009, the incidence rate and HR of psoriasis increased according to the number of MetS components in 2012.

## Discussion

In this large-scale nationwide population-based study, we calculated the risk of psoriasis based on MetS diagnosis changes over a period of 4 years. Our results showed that the risk of psoriasis in the pre-MetS group was similar to that in the control group; however, the risk was greater in the post-MetS and continuous-MetS groups than in the control group. This tendency was slightly different for each component of MetS. First, for the waist circumference criterion, the risk of psoriasis was higher in all three groups (pre-MetS, post-MetS, and continuous-MetS) than in the control group. For TG and HDL cholesterol criteria, the risk of psoriasis was increased only in the post-MetS and continuous-MetS groups. For the fasting blood glucose criterion, the risks of psoriasis were similar in all groups. Finally, for the blood pressure criterion, the risk of psoriasis decreased in the pre-MetS and continuous-MetS groups.

Many reports have studied the relationship between psoriasis and MetS, but most of those studies were designed to evaluate the risk of MetS in patients with psoriasis^6,15–23^. To our knowledge, there have been few reports concerning the risk of psoriasis in patients with MetS^9,10,24^. One such report described a large-population based cross-sectional study involving 34,996 individuals, which was published by a Norwegian research team in 2018^10^. The relative risk of psoriasis in the individuals with MetS was 1.66 compared with the control group. They also examined the associations of each MetS criterion with development of psoriasis and found increased risks of psoriasis in patients with waist circumference, TG, and HDL components but not in those with high blood pressure or blood glucose level. Those results are consistent with our findings.

Our study differs from others in that the patients with MetS in our sample were followed for a period of 4 years and were divided into four groups for systematic analysis of the associations between psoriasis and MetS components. In the pre-MetS group, we found similar risks of psoriasis in the control group, but the post-MetS and continuous-MetS groups had significantly higher risks of psoriasis than the control group. This trend was consistent for the waist circumference and TG MetS components. For the HDL criterion, the HR was higher in the post-MetS and continuous-MetS group than in the pre-MetS group. This implies that components of MetS, particularly obesity and dyslipidemia, influence the development of psoriasis.

The association between psoriasis and MetS has been reported, but it is unclear whether one of the two conditions precedes or induces the other^10^. Central obesity and dyslipidemia contribute to increased risk of psoriasis. Several hypotheses have been suggested to explain the increased risk of psoriasis in individuals with MetS. A convincing hypothesis is that the increasing levels of inflammatory markers in MetS lead to increased risk of psoriasis. MetS and psoriasis share inflammatory pathways, such as the T helper‐17‐mediated or T helper-1-mediated inflammation pathways^3^. Many similar cytokines contribute to the pathogeneses of psoriasis and impaired lipoprotein regulation^25,26^. Moreover, patients with central obesity have excessive adipose tissue, which secretes adipokines, drivers of various chronic inflammation processes such as psoriasis^27^. Another hypothesis states that the two diseases share genetic loci. One study demonstrated shared genes between psoriasis and dyslipidemia, hypertension, and coronary artery disease^28^, but other studies have found no genetic associations between psoriasis and MetS or coronary artery disease^28–31^. Our finding that risk of psoriasis was decreased in the pre-MetS group supported the hypothesis that persistently elevated inflammatory markers in MetS lead to an increased risk of psoriasis. The inflammation in MetS differs from the traditional concept of tumor, redness, pain, and heat. It is helpful to characterize MetS inflammation as “low-grade” or chronic. The condition is caused primarily by nutrient and metabolic excesses, which trigger various pathologic mechanisms (e.g., Toll-like receptor pathways or inflammasomes such as NLRP3 activation^32^) that also are increased in psoriasis^33,34^. Therefore, we infer that the continually increased inflammation in MetS plays a role in the development of psoriasis.

Additionally, we analyzed the associations of MetS components with risk of psoriasis. For the waist circumference component, the HR in the continuous-MetS group was 1.146 (95% CI, 1.097–1.197), the strongest association of all MetS components. This was followed by the associations of HDL (HR, 1.106; 95% CI, 1.068–1.145) and TG (HR, 1.013; 95% CI, 0.982–1.046). The fasting glucose component did not show a significant association with risk of psoriasis, and the blood pressure component was associated with lower risk in both the pre-MetS and continuous-MetS groups compared with the control group. These results differ from those of other studies, which showed positive associations between risk of psoriasis and all MetS components^9,22,35,36^. Other recent, large, well-designed studies showed that dyslipidemia and abdominal obesity were the main MetS components positively associated with risk of psoriasis; however, they found no significant associations of the MetS components of blood pressure and fasting glucose level with risk of psoriasis^10,15,19–21,23,37^, consistent with our results.

The strength of our study lies in its large cohort size, which was representative of the entire Korean population; therefore, the findings likely are generalizable to the general public in Korea. In addition, by dividing the MetS patients into four groups based on changes in MetS components, we believe that our results contribute to a more detailed and better understanding of the relationship between MetS and psoriasis. However, there are some notable limitations of this study. First, because the diagnoses of MetS and psoriasis was dependent on ICD-codes, coding or misclassification errors might have been included. Still, a study verifying the frequency of such errors found that approximately 70% of the diagnostic codes were consistent with those in the medical records^11^. Second, this study lacked information on genetic factors, which constitute the greatest influence on psoriasis; and drug history, such as that of lithium and interferon, which are well-known drugs for risk of developing psoriasis^2^. Nevertheless, we corrected for well-known confounding factors associated with psoriasis such as smoking, alcohol consumption, and BMI. Third, this study included only psoriasis patients who had received prescription medications and treatments, including phototherapy and topical agents, from physicians or dermatologists after being diagnosed with psoriasis. Some patients with psoriasis might have been omitted, especially those with mild disease. However, many epidemiological studies in Korea have been conducted using claims data due to the accessibility of these records in Korea and accuracy of Korean National Health Insurance data^38,39^.

## Conclusions

Our findings from this population-based nationwide study in Korea demonstrated the effects of changes in MetS components on risk of psoriasis. Our study differs from prior investigations because we focused on MetS diagnosis changes, and our results can help to better elucidate the association between psoriasis and MetS. Remarkably, the risk of psoriasis was higher in patients with continuous- or post-MetS than in those without MetS (regardless of prior MetS status). Therefore, we emphasize the need to explain these findings to patients.

## Data Availability

The data used in this study are owned by the Korean National Health Insurance Service, and public sharing of these data is restricted. The authors accessed these data after requesting them from the National Health Insurance Data Sharing Service (NHISS). The authors of this study enjoyed no special access privileges before requesting the data, and they confirm that other qualified researchers should be able to request access to these data from the NHISS. For more information about this process, please see https://nhiss.nhis.or.kr/bd/ab/bdaba032eng.do.

## References

[1] Michalek IM, Loring B, John SM (2017). A systematic review of worldwide epidemiology of psoriasis. J. Eur. Acad. Dermatol. Venereol..

[2] Armstrong AW, Read C (2020). Pathophysiology, clinical presentation, and treatment of psoriasis: A review. JAMA.

[3] Davidovici BB (2010). Psoriasis and systemic inflammatory diseases: Potential mechanistic links between skin disease and co-morbid conditions. J. Invest. Dermatol..

[4] Chat VS, Uppal SK, Kearns DG, Han G, Wu JJ (2021). Translating the 2020 AAD-NPF guidelines of care for the management of psoriasis with systemic nonbiologics to clinical practice. Cutis.

[5] Eckel RH, Grundy SM, Zimmet PZ (2005). The metabolic syndrome. Lancet.

[6] Grundy SM, Brewer HB, Cleeman JI, Smith SC, Lenfant C (2004). Definition of metabolic syndrome: Report of the National Heart, Lung, and Blood Institute/American Heart Association conference on scientific issues related to definition. Arterioscler. Thromb. Vasc. Biol..

[7] Alberti KG (2009). Harmonizing the metabolic syndrome: A joint interim statement of the International Diabetes Federation Task Force on Epidemiology and Prevention; National Heart, Lung, and Blood Institute; American Heart Association; World Heart Federation; International Atherosclerosis Society; and International Association for the Study of Obesity. Circulation.

[8] Armstrong AW, Harskamp CT, Armstrong EJ (2013). Psoriasis and metabolic syndrome: A systematic review and meta-analysis of observational studies. J. Am. Acad. Dermatol..

[9] Kim HN, Han K, Park YG, Lee JH (2019). Metabolic syndrome is associated with an increased risk of psoriasis: A nationwide population-based study. Metabolism.

[10] Snekvik I, Nilsen TIL, Romundstad PR, Saunes M (2019). Metabolic syndrome and risk of incident psoriasis: Prospective data from the HUNT Study, Norway. Br. J. Dermatol..

[11] Song SO (2014). Background and data configuration process of a nationwide population-based study using the Korean national health insurance system. Diabetes Metab. J..

[12] Grundy SM (2005). Diagnosis and management of the metabolic syndrome: An American Heart Association/National Heart, Lung, and Blood Institute Scientific Statement. Circulation.

[13] Lee SY (2007). Appropriate waist circumference cutoff points for central obesity in Korean adults. Diabetes Res. Clin. Pract..

[14] Wen CP (2009). Are Asians at greater mortality risks for being overweight than Caucasians? Redefining obesity for Asians. Public Health Nutr..

[15] Gisondi P, Fostini AC, Fossà I, Girolomoni G, Targher G (2018). Psoriasis and the metabolic syndrome. Clin. Dermatol..

[16] Cohen AD, Sherf M, Vidavsky L, Vardy DA, Shapiro J, Meyerovitch J (2008). Association between psoriasis and the metabolic syndrome. Dermatology.

[17] Langan SM (2012). Prevalence of metabolic syndrome in patients with psoriasis: A population-based study in the United Kingdom. J. Invest. Dermatol..

[18] Nisa N, Qazi MA (2010). Prevalence of metabolic syndrome in patients with psoriasis. Indian J. Dermatol. Venereol. Leprol..

[19] Love TJ, Qureshi AA, Karlson EW, Gelfand JM, Choi HK (2011). Prevalence of the metabolic syndrome in psoriasis: Results from the national health and nutrition examination survey, 2003–2006. Arch. Dermatol..

[20] Meziane M (2016). Metabolic syndrome in Moroccan patients with psoriasis. Int. J. Dermatol..

[21] Mebazaa A (2011). Metabolic syndrome in Tunisian psoriatic patients: prevalence and determinants. J. Eur. Acad. Dermatol. Venereol..

[22] Milčić D (2017). Prevalence of metabolic syndrome in patients with psoriasis: A hospital-based cross-sectional study. An. Bras. Dermatol..

[23] Danielsen K (2015). Elevated odds of metabolic syndrome in psoriasis: A population-based study of age and sex differences. Br. J. Dermatol..

[24] Praveenkumar U, Ganguly S, Ray L, Nanda SK, Kuruvila S (2016). Prevalence of metabolic syndrome in psoriasis patients and its relation to disease duration: A hospital based case-control study. J. Clin. Diagnost. Res..

[25] Armstrong EJ, Krueger JG (2016). Lipoprotein metabolism and inflammation in patients with psoriasis. Am. J. Cardiol..

[26] Ferretti G (2012). Correlation between lipoprotein(a) and lipid peroxidation in psoriasis: Role of the enzyme paraoxonase-1. Br. J. Dermatol..

[27] Hjuler KF (2017). Increased global arterial and subcutaneous adipose tissue inflammation in patients with moderate-to-severe psoriasis. Br. J. Dermatol..

[28] Lu Y (2013). Association of cardiovascular and metabolic disease genes with psoriasis. J. Invest. Dermatol..

[29] Koch M (2015). Psoriasis and cardiometabolic traits: modest association but distinct genetic architectures. J. Invest. Dermatol..

[30] Gerdes S, Osadtschy S, Buhles N, Baurecht H, Mrowietz U (2014). Cardiovascular biomarkers in patients with psoriasis. Exp. Dermatol..

[31] Gupta Y (2013). Genetic control of psoriasis is relatively distinct from that of metabolic syndrome and coronary artery disease. Exp. Dermatol..

[32] Hotamisligil GS (2006). Inflammation and metabolic disorders. Nature.

[33] Gaire BP (2020). Lysophosphatidic acid receptor 5 contributes to imiquimod-induced psoriasis-like lesions through NLRP3 inflammasome activation in macrophages. Cells.

[34] Irrera N (2017). BAY 11–7082 inhibits the NF-κB and NLRP3 inflammasome pathways and protects against IMQ-induced psoriasis. Clin. Sci. (Lond.).

[35] Madanagobalane S, Anandan S (2012). Prevalence of metabolic syndrome in South Indian patients with psoriasis vulgaris and the relation between disease severity and metabolic syndrome: A hospital-based case-control study. Indian J. Dermatol..

[36] Miller IM (2015). The association of metabolic syndrome and psoriasis: a population- and hospital-based cross-sectional study. J. Eur. Acad. Dermatol. Venereol..

[37] Albareda M (2014). Metabolic syndrome and its components in patients with psoriasis. Springerplus.

[38] Han JH (2018). Epidemiology and medication trends in patients with psoriasis: A nationwide population-based cohort study from Korea. Acta Dermatol. Venereol..

[39] Lee JY, Kang S, Park JS, Jo SJ (2017). Prevalence of psoriasis in Korea: A population-based epidemiological study using the Korean National Health Insurance Database. Ann. Dermatol..

